# Correction to “Surface Functionalization of Hydroxyapatite Scaffolds with MgAlEu‐LDH Nanosheets for High‐Performance Bone Regeneration”

**DOI:** 10.1002/advs.76078

**Published:** 2026-06-17

Guanyun Wang, Zehui Lv, Tao Wang, Tingting Hu, Yixin Bian, Yu Yang, Ruizheng Liang, Chaoliang Tan, Xisheng Weng, “Surface Functionalization of Hydroxyapatite Scaffolds with MgAlEu‐LDH Nanosheets for High‐Performance Bone Regeneration,” *Advanced Science* 10, no. 1 (2023): e2204234, https://doi.org/10.1002/advs.202204234.

In the originally published article, an incorrect Alizarin Red S staining image of MC3T3‐E1 in the HL8 group (Figure 2b) was used (originating from the HL16 group). This has now been corrected, and the revised Figure 2 is provided below. The corrected data were derived from the original experiments, and no new experiments were performed. The results and conclusions remain unchanged.

**Figure:**
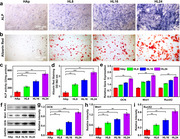
**FIGURE 2** | The osteogenic properties of HAp/MAE‐LDH scaffolds. (a, b) Optical microscope images of Alkaline phosphatase staining and Alizarin red S staining of MC3T3‐E1 cultured with HAp, HL8, HL16, and HL24 scaffolds. Scale bar: 200 µm. (c, d) Quantitative analysis of Alkaline phosphatase staining and Alizarin red S staining using ImageJ 1.52q1.52v software. (e) qPCR results detecting the osteogenic gene (OCN, Wnt1, and RunX2) expression of MC3T3‐E1 cultured with HAp, HL8, HL16, and HL24 scaffolds. (f) Western blot results detecting the osteogenic proteins (OCN, Wnt1, and RunX2) expression of MC3T3‐E1 cultured with HAp, HL8, HL16, and HL24 scaffolds. (g–i) Quantitative analysis of the Western blot results evaluating osteogenic proteins expression of MC3T3‐E1 using ImageJ 1.52q1.52v software. Data are presented as mean values ± s.d. (*n* = 3). ^**^ *p* < 0.01, ^***^ *p* < 0.001 (one‐way ANOVA).

In the Supporting Information, two image errors were identified: the Calcein‐AM/PI (live/dead) staining image of MC3T3‐E1 in the HL16 group (Figure S7a, Day 5), which originated from the HL8 group (Day 5), and the optical microscope image of HUVECs in the HAp/MgAl‐LDH group (Figure S13a), which originated from the HAp/MAE‐LDH group. These have been corrected, and the revised Figures S7 and S13 are provided below. The corrected data were derived from the original experiments, with no new experiments performed. The results and conclusions remain unchanged.

**Figure:**
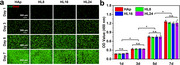
**FIGURE S7** | (a) Calcein‐AM/PI (live/dead) staining and (b) CCK‐8 assay results evaluating the biocompatibility of HAp, HL8, HL16, and HL24 scaffolds for MC3T3‐E1 up to the 7th day. Data are presented as mean values ± s.d. (*n* = 4). ^*^ *p* < 0.05 (one‐way ANOVA).

**Figure:**
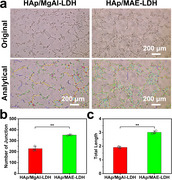
**FIGURE S13** | Matrigel experiment results and further quantitative analysis results (ImageJ 1.52q1.52v software) accessing the vessel generation capability of HUVECs cultured with HAp/MgAl‐LDH or HAp/MAE‐LDH. Data are presented as mean values ± s.d. (*n* = 3). ^**^ *p* < 0.01 (one‐way ANOVA).

We apologize for this error.

